# LiteFer: An Approach Based on MobileViT Expression Recognition

**DOI:** 10.3390/s24185868

**Published:** 2024-09-10

**Authors:** Xincheng Yang, Zhenping Lan, Nan Wang, Jiansong Li, Yuheng Wang, Yuwei Meng

**Affiliations:** Electronic Information Department, Dalian Polytechnic University, Dalian 116034, China; 220520854000556@xy.dlpu.edu.cn (X.Y.); lanzp@dlpu.edu.cn (Z.L.); 220510811000489@xy.dlpu.edu.cn (N.W.); 220520854000572@xy.dlpu.edu.cn (J.L.); 220510811000499@xy.dlpu.edu.cn (Y.W.)

**Keywords:** Facial expression recognition, Deep learning, Lightweight network

## Abstract

Facial expression recognition using convolutional neural networks (CNNs) is a prevalent research area, and the network’s complexity poses obstacles for deployment on devices with limited computational resources, such as mobile devices. To address these challenges, researchers have developed lightweight networks with the aim of reducing model size and minimizing parameters without compromising accuracy. The LiteFer method introduced in this study incorporates depth-separable convolution and a lightweight attention mechanism, effectively reducing network parameters. Moreover, through comprehensive comparative experiments on the RAFDB and FERPlus datasets, its superior performance over various state-of-the-art lightweight expression-recognition methods is evident.

## 1. Introduction

Expression recognition has become a focal point in computer vision and artificial intelligence research, experiencing significant growth and application in recent years. Advances in computing power and the emergence of deep learning have propelled expression recognition into various fields. Emotions, crucial for human communication, carry a wealth of information, leading expression recognition beyond emotion analysis to encompass domains such as social media, healthcare, education, security monitoring, and game development.

Expression-recognition methodologies are broadly classified into two categories: traditional and deep learning-based approaches. Traditional methods are reliant on handcrafted features and frequently operate within controlled dataset environments. Features extracted from specific facial landmarks, such as the nose, mouth and eyes, must exhibit consistency throughout the entirety of the image. Common traditional techniques include the histogram of orientation gradients (HOG) [1], Gabor wavelet transform [2], and local binary patterns (LBPs) [3]. Traditional methods can effectively handle expression recognition with limited data; however, they struggle with larger datasets due to manual feature extraction, an inability to capture deep features, and susceptibility to factors such as variations in illumination and occlusion. This article’s proposed model is an enhancement of MobileViT, incorporating the GAM attention mechanism and CB broadcasting mechanism while reducing FLOPs. Compared to existing models with large parameter counts, it achieves performance comparable to traditional large models with minimal computational resources. Additionally, it demonstrates significant advantages over current lightweight models.

As deep learning methodologies rapidly advance in computer vision, researchers have proposed various methods to improve the performance of convolutional neural networks for facial-expression recognition tasks. Among them, Luan [4] et al. proposed a residual masking network that combines a deep residual network and a U-Net-like architecture. This neural network employs a segmentation network to enhance the feature map, prioritizing pertinent information to enhance recognition precision. Yao et al. [5] introduced HoloNet, a CNN-based network that combines a residual network with the CReLu activation function, increasing the network’s depth and width to enhance performance. Dosovitskiy et al. introduced the ViT model; this method surpasses convolutional neural networks (CNNs) and finds extensive applications in various computer vision tasks. Xiuwen Lu [6] et al. proposed a two-branch hybrid residual network that combines an adaptive feature-fusion module and an R_GHOST layer to enhance feature extraction and reduce network parameters. In addition, Yu Zhou [7] et al. combined a quaternion orthogonal transformer with a vision transformer (ViT) model to achieve superior results and reduce computation on multiple datasets. Liu, Shu [8] et al. proposed a dual-branch adaptive distribution fusion (Ada-DF) framework, where an auxiliary branch is constructed to obtain the label distributions of the samples. Then, the class distributions of emotions are computed through the label distributions of each emotion to eliminate the ambiguity present in the distributions. Finally, these two distributions are adaptively fused based on attention weights to train the target branch. Extensive experiments have been conducted on three real-world datasets (RAF-DB, AffectNet, and SFEW), where the Ada-DF demonstrates superior performance compared to existing state-of-the-art methods. Huy-Hoang Dinh [9] et al. proposed the FGW method, which includes an expansion part, a depth-wise part, and a classifier part. Additionally, it employs regularization techniques such as batch normalization and dropout to enhance the model’s learning efficiency. The FGW achieves excellent results on the RAFDB and FERPlus datasets with a minimal number of parameters. However, these methods do not achieve a good balance between lightweight design and accuracy. Thus, in this paper, an improved model called LiteFer is proposed, which builds upon classical models and the methods mentioned above, and the primary contributions are as follows:

(1) Adding a GAM attention mechanism module [10] to the MV2 structure in LiteFer to improve the network performance without increasing the network complexity too much.

(2) Adding an adaptive token sampling module [11] to the ViT structure in LiteFer can effectively reduce GFlops and lower the computational cost.

(3) Adding the up–down broadcast module (CB) [12] at the end of the MLP in the LiteViT structure of LiteFer can effectively improve the ViT performance.

## 2. Related Work

### 2.1. Attention Mechanisms

Attention mechanisms are pivotal components in deep learning methodologies, serving a crucial function. They are utilized to augment the model’s concentration on the input data, consequently enhancing the performance and generalization capability of the model. Among them, the SE attention mechanism was proposed by Hu [13] et al. in 2018. This mechanism enriches the model’s discernment of distinct channels through the acquisition of weights between them. This adaptation enables the network to better capture the channel correlations present in the input data, consequently enhancing the model’s performance. Wang [14] et al. introduced the ECA attention mechanism, which utilizes a local cross-channel interaction strategy that is employed without diminishing dimensionality. This approach effectively prevents the negative effects of reducing feature-map dimensionality on channel attention learning, thereby enhancing the model’s performance and reducing complexity. In order to address the limitations of traditional convolutional neural networks in handling information of varying scales, shapes, and orientations, Sanghyun Woo [15] et al. proposed a model that integrates channel and spatial attention modules to enhance feature representation by element-wise multiplication, thus enhancing the model’s performance. Subsequently, Yichao Liu [10] and others proposed the global average pooling (GAM) attention mechanism; this process captures the global contextual information of the entire input by employing a global average pooling operation. Indeed, this capability enables the model to grasp the comprehensive relationship and subsequently enhance its performance even further. The development and application of these attention mechanisms provide more powerful modeling capabilities for deep learning models. Variants such as ECA and GAM offer valuable insights for designing lighter and more efficient attention mechanisms.

### 2.2. Depth-Separable Convolution

Depth-separable convolution was first introduced by Howard et al. [16] in their 2017 paper. They utilized depth-separable convolution to reduce the model size for situations with limited computational resources on mobile devices. Unlike traditional convolution, deep convolution, also known as depth-wise convolution in the context of depth-separable convolution, performs convolution operations on only one channel of the input image per convolution kernel. This process is repeated for each channel independently, before combining the results through a pointwise convolution to generate the final output, and the number of convolution kernels matches the number of channels in the input image. Point-by-point convolution, also referred to as 1 × 1 convolution, constitutes a distinctive instance within regular convolution. Specifically, when employing a convolution kernel size of 1 × 1, standard convolution undertakes convolution operations across the entirety of the input image, considering all channels simultaneously. It applies a single convolutional filter of size 1 × 1 to each location in the input volume, resulting in a linear combination of the input channels. This operation is commonly used for dimensionality reduction or feature transformation, as it helps adjust the depth of the feature maps while preserving spatial information. Point-by-point convolution does not change the size of the feature map, only the number of channels of the feature map. The channels of the input feature map are first convolved using deep convolution to obtain the corresponding channel features, and then the extracted channel features are combined using pointwise convolution. Depth-separable convolution has been used not only for lightweight models but also in many other areas, where it has excelled in different tasks such as semantic segmentation, target detection, and image classification. Researchers have further enhanced the performance of depth-separable convolution by incorporating attention mechanisms, skip connections, and other techniques.

### 2.3. ViT

The recent advancements of deep learning in computer vision, primarily through convolutional neural networks (CNNs), has significantly advanced tasks such as semantic-segmentation image classification and target detection. Traditional convolutional structures, however, face challenges in handling global information and struggle with large-size images. To overcome these limitations, the vision transformer (ViT) model, proposed by Dosovitskiy et al. [17], introduces a global context-modeling approach based on self-attention mechanisms. This allows for the efficient learning of image representations without relying on convolution.

The introduction of ViT has sparked extensive research into the application of attention mechanisms in computer vision. Researchers have explored various aspects, such as different attention head designs, the number of layers in the block, strategies for chunking input images, and other enhancements. Additionally, hybrid architectures that combine convolutional layers with attention mechanisms have been developed to enhance model performance. For instance, MobileViT [18] integrates MobileNet with ViT to address challenges such as training difficulties and biased induction when using ViT alone. This integration has led to achieving state-of-the-art results on datasets like RAFDB and FERPlus.

The success of ViT has not only revolutionized computer vision tasks but has also inspired exploration into full-attention modeling across other domains. This showcases the versatility and powerful modeling capabilities of attention mechanisms. The emergence of ViT marks a significant milestone, not just in image classification but also in paving the way for the widespread adoption of attention mechanisms in deep learning. This opens up exciting new research avenues and possibilities for leveraging attention mechanisms in various applications beyond computer vision.

## 3. Modeling Framework

### 3.1. Overall Structure

#### 3.1.1. Design of the LiteFer

**Figure 1:**
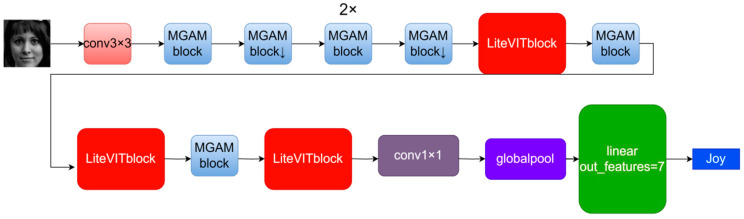
LiteFer overall structure.

First, a 224 × 224 facial image is input into the network, which undergoes downsampling through a 3 × 3 convolution. This is followed by a series of MGAM blocks and LiteViT blocks for changing the number of output channels and further downsampling (as indicated by ↓ in Figure 1 for downsampling functionality). Finally, a 1 × 1 convolution is applied, followed by global pooling, and a linear layer is used to obtain the classification result. The structure is illustrated in Figure 1. The specific details of LiteFer can be found in Table 1.

#### 3.1.2. Model Hierarchy

The hierarchical structure of the model is clearly divided into layers, each with a specific function. Through information transfer between the layers, the model can gradually extract more abstract features. The final global representation is generated through multiple transformer encoders to grasp extensive interconnections within images.

#### 3.1.3. Output Layer

The model’s final output is generated by passing through a global average pooling layer, followed by mapping to a predefined number of categories using a fully connected layer. This output layer is designed to maintain the lightweight nature of the model while ensuring the effective categorization of the input images.

### 3.2. MGAMblock

#### 3.2.1. General Structure of MGAMblock

**Figure 2:**
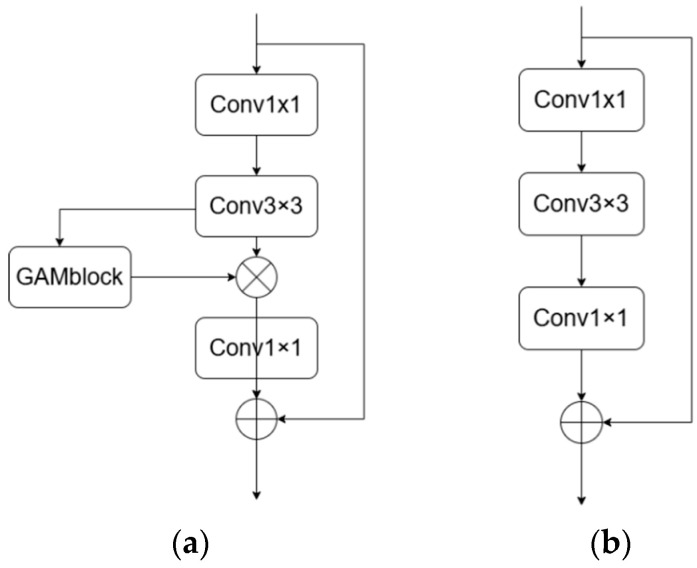
Comparison of MGAMblock and MV2block. (**a**) MGAM block. (**b**) MV2 block.

This module applies the inverse residual structure proposed by MV2 [19], where the input features are first subjected to 1 × 1 pointwise convolution to enhance the feature dimension. Subsequently, following a 3 × 3 deep convolutional operation for feature extraction, the GAM attention mechanism module is added (as shown in Figure 2). This addition effectively addresses the negative impact of feature-map dimensionality reduction on channel attention learning. It enhances the model’s attention on target features while concurrently reducing model complexity. The dimensionality is standardized by 1 × 1 convolution, and all components utilize the SILU activation function [20]. Figure 2 shows the model diagrams of MV2 and MGAM. MGAM is used in the LiteFer method proposed in this paper, while MV2 is employed in MobileViT.

#### 3.2.2. GAM Attention Mechanism

**Figure 3:**
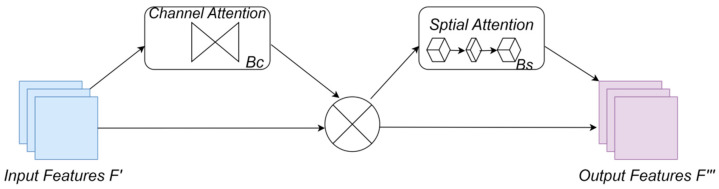
GAM attention mechanism.

The GAM attention mechanism [10] is similar to the CBAM attention mechanism [15] in that it also uses spatial and channel attention mechanisms. The difference lies in the treatment of these two attention mechanisms, and the GAM attention mechanism is shown in Figure 3. Given the input feature mapping of the GAM attention mechanism, the intermediate states and outputs are outlined as follows:

(1) F″=Bc(F′)⊗F′

(2) F‴=Bc(F″)⊗F″

where *B_c_* and *B_s_* represent the channel attention and spatial attention modules, respectively, and ⊗ denotes multiplication by elements.

**Figure 4:**
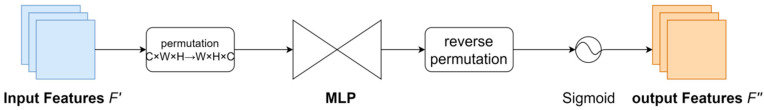
Channel attention sub-module.

The channel attention sub-module utilizes a three-dimensional layout to preserve information across three dimensions. It then strengthens cross-dimensional channel-space dependencies through a two-layer MLP (multilayer perceptron). The channel attention mechanism aims to learn the importance of each channel and adaptively recalibrate them across different spatial locations. This is achieved by learning channel-wise attention weights that scale the feature responses within each channel. By emphasizing informative channels and suppressing less relevant ones, the model can effectively enhance feature discrimination and representation. The channel attention sub-module is depicted in Figure 4.

**Figure 5:**
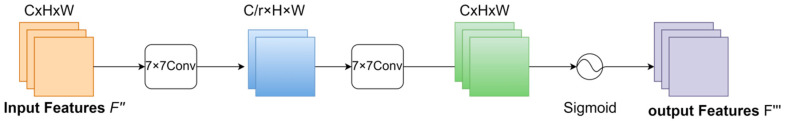
Spatial attention sub-module.

Within the spatial attention sub-module, two convolutional layers are employed to integrate spatial information for highlighting spatial details, Spatial attention in deep learning refers to a mechanism where the model focuses on specific spatial locations of an input. It allows the model to weight different parts of the input differently based on their relevance to the task. This is particularly useful in tasks such as image classification or object detection. In the channel attention sub-module, the same reduction ratio as in the CBAM is utilized, denoted as (r). However, a drawback arises from the maximum pooling operation, which reduces the utilization of information. Here, the pooling operation is excluded to more effectively preserve the feature mapping. The spatial attention sub-module is depicted in Figure 5.

### 3.3. LiteViT Block

#### 3.3.1. LiteViT Block Overall Structure

**Figure 6:**
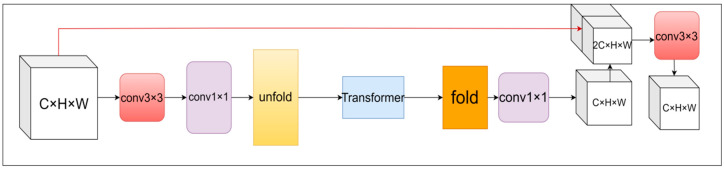
LiteViT block.

The LiteViT block consists of an extended convolutional layer, a depth-separable convolutional layer, an ATS module, a transformer encoder, and a CB module. These components collaborate to heighten sensitivity to local features and facilitate the capture of global information via the attention mechanism. The outcome of the LiteViT block is a fused representation of both local and global features. The feature map is initially processed locally using a convolutional layer with a 3 × 3 convolutional kernel size. The number of channels is then adjusted by a separate convolutional layer with a 1 × 1 convolutional kernel size, allowing for precise control over the output dimensions and facilitating feature extraction. The global feature modeling is then performed through the Unfold -> Transformer -> Fold structure. The self-attention mechanism within the transformer module integrates the ATS module [11], and the CB [12] module is added at the end of the MLP layer. Subsequently, the number of channels is returned to its original size using a 1 × 1 convolutional layer, ensuring that the output feature map maintains the same number of channels as the input feature map. The shortcut branch and the original input feature map are concatenated (spliced along the channel direction), and finally, the output is obtained by a convolutional layer with a convolutional kernel size of 3 × 3. The overall structure of the LiteViT block is shown in Figure 6.

#### 3.3.2. ATS Module

(1) Token Scoring.

**Figure 7:**
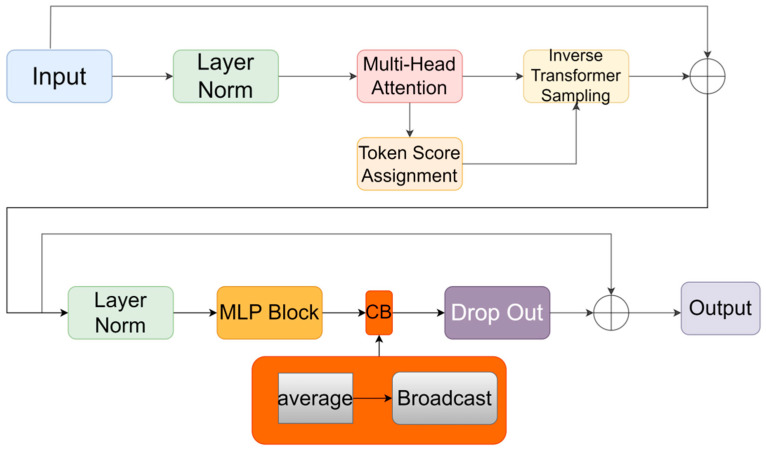
Transformer encoder.

The ATS (adaptive training sampler) module, as described in reference [11], is a parameter-free differentiable module designed for efficiently sampling input tokens. In this paper, the ATS module functions by initially assigning significance scores to N input markers, followed by the selection of a subset based on these scores. By imposing a maximum limit K on the number of markers, an upper bound on the FLOPs is established. During the sampling process, some input markers may be sampled multiple times, but only one instance of each marker is retained. Consequently, the number of sampled markers K′ typically ends up being lower than K. Figure 7 illustrates a schematic depiction of how the ATS module is integrated into the transformer structure.

Token score assignment and inverse transformer sampling, as shown in Figure 7, together make up the ATS module. When it comes to the input markers $I\in R^{(N+1)\times d}$ of the self-attention layer, ViT pre-processes them by adding a classification marker to the input markers before passing them to the model. This classification marker is located in the first position of each block and produces the corresponding output marker in the final transformer block for the calculation of the classification probability. Although the classification markers are retained, the goal of this paper is to dynamically adjust the number of output markers O ∈ $R^{(k^{\prime }+1)\times d}$ . The condition K′ ≤ K ≤ N ensures that the parameter (K) controls the maximum number of markers to be sampled, where (K) is a parameter. In the standard attention layer, the query Q ∈ R (N + 1) × d is computed using the input tokens I ∈ R (N + 1) × d, the key K ∈ R (N + 1) × d and the value V ∈ R (N + 1) × d to derive the attention matrix ( A ):

(3) $$A=Soft\max(\frac{{QK}^{T}}{\sqrt{d}})$$

As a result of the function Softmax , each row of the attention matrix $A\in R^{\left(N+1)\times (N+1\right)}$ sums to 1. The output token O is then computed by using a combination of the attention weights plus the weighted values.

(4) O=AV

Each row of matrix ( A ) exactly represents the attention weights assigned to each input marker (or token) with respect to the output markers. These weights represent the significance or contribution of each input feature to the generation or determination of the output features, reflecting the importance or relevance of each input marker in the context of producing the output. This mechanism enables the model to selectively attend to different parts of the input sequence when generating the output, enabling it to capture relevant information and make informed decisions. Since $A_{1,:}$ contains the attentional weights of the categorized markers and $A_{1,j}$ denotes the importance of the output categorized markers to the input marker j, this paper uses $A_{1,2}\ldots \ldots A_{1,N+1}$ . As pruning the attention matrix A produces the importance score S, the j th marker importance score is given by

(5) $$S_{j}=\frac{A_{1,j}\times \left\|V_{j}\right\|}{\sum_{i=2}A_{1,j}\times \left\|V_{j}\right\|}$$

Included among these is i,j∈{2,N} . For the multi-head self-attention layer, a score is computed for each head, and the scores are then summed.

(2) Token Sampling.

Following the computation of importance scores for all markers, removing their corresponding rows from the attention matrix ( A ) is an option. Typically, a straightforward method involves selecting the top (K) markers based on their importance scores. However, empirical evidence shows that this approach may not effectively adapt to selecting (K′) markers where (K′ \leq K), as it disregards markers with lower scores entirely. Nonetheless, retaining some of these lower-scoring markers might be beneficial, particularly in scenarios where features lack discrimination. For example, in the early stage, the attention weight of multiple markers with similar keys may be reduced due to the Softmax function. Although one of the markers may be important in later stages, selecting the first K markers may discard them all. Thus, it is suggested to select sample markers based on their importance scores. In this scenario, the probability of sampling a similar marker equals the sum of their scores. Additionally, the proposed sampling approach favors selecting more markers in the early stages compared to the later stages. For the sampling procedure, this paper employs inverse permutation sampling, where markers are sampled according to their importance score ( S (5)). As the scores are normalized, they can be interpreted as probabilities, and the cumulative distribution function ( CDF ) of ( s ) can be computed.

(6) $$CDF_{i}=\sum_{j=2}^{j=1}S_{j}$$

The sampling function is derived by taking the inverse function of the cumulative distribution function CDF :

(7) $$\gamma (k)=CDF^{-1}(k)$$

k∈[0,N] . In other words, the importance score is utilized to compute a mapping function of the indexes between the original markers and the sampled markers in a scientific context. To obtain k samples, one can perform k sampling iterations from a uniform distribution U[0,1] . While randomization may be suitable for certain applications, deterministic approaches are often preferred. Hence, a fixed sampling scheme is employed in both training and inference, where a specific value of k={2/k,2/3k,…,(2k−1)/2k} is chosen. Using the indices of the sampled tokens, the attention matrix A∈R(N+1)×(N+1) is optimized by selecting the rows that correspond to the sampled $K^{\prime }+1$ tokens. In this paper, $A^{s}\in R(K^{\prime }+1)\times (N+1)$ is used to represent the refined attention matrix. To obtain the output labeling $o\in (K^{\prime }+1)\times d$ , we replace the attention matrix A in (3) with the refined matrix $A_{s}$ :

(8) $$O=A^{s}V$$

Finally, these outputs are labelled as inputs for the next stage.

#### 3.3.3. CB Module

Nam Hyeon-Woo [12] et al. found that additional spatial interactions can bring performance gains to ViT models and the proposed broadcast context module ( CB ), which does not add additional parameters to the model by the following operation:

Given a sequence containing N tokens, the CB module reinjects the average pooled tokens into the tokens in the following way:

(9) $$CB(X_{i})=\frac{X_{i}+\frac{1}{N}\sum_{j=1}^{N}X_{j}}{2}\;for\;every\;Token\;i$$

where $X_{i}$ ∈ R and d is the i th token in X. Figure 4 illustrates the CB module. The CB module is located at the end of the multilayer perceptron (MLP) block. As shown in Figure 7, the CB module in this paper is placed at the end of the ViT architecture. Nam Hyeon-Woo [12] et al. demonstrated that the performance improvement is most significant when the CB is inserted after the MLP block.

### 3.4. LiteFer Details Table

Details of LiteFER are shown in Table 1, where ↓ indicates downsampling.

**Table 1:** The detailed LiteFer configuration.

Layers	Size	Output Stride	Repetition	Output Channels
Image	224 × 224	1		
Conv3 × 3 ↓			1	
MGAM block	112 × 112	2	1	16
MGAM block ↓			1	
MGAM block	56 × 56	2	2	24
MGAM block ↓			1	
LiteViT	28 × 28	2	1	48
MGAM block ↓			1	
LiteViT	14 × 14	2	1	64
MGAM block ↓			1	
LiteViT	7 × 7	2	1	80
Conv1 × 1			1	320
Global pool	1 × 1	2	1	1000
Linear				

## 4. Experimental Setup

### 4.1. Environment Setup

In this paper, the training machine of the model is equipped with an NVIDIA RTX3060ti graphic processing unit (GPU), which has 4864 CUDA units and 8 GB of video memory capacity, using a GDDR6X video memory type. The central processing unit (CPU) is an Intel Core i5-12490F at 3 GHZ, and the experimental operating system is 64-bit Windows using the Pytorch deep learning framework. Furthermore, 224 × 224 RGB image data are used as the input, the batch size is set to 8, the initial learning rate is set to 0.0002, the training time is 200 h, and 200 rounds of training are performed.

### 4.2. Introduction of Dataset

In order to validate the model’s performance, this paper conducts an experimental evaluation of the model based on the RAFDB and FERPlus datasets and a natural face expression dataset.

The RAFDB dataset [21], abbreviated as RAFDB, presents a collection of authentic facial expressions. It showcases images capturing a wide array of facial emotions with intricate details. The dataset includes various facial expressions categorized into seven distinct classes: Surprise, Fear, Disgust, Joy, Sadness, Anger, and Neutral. It boasts a total of 12,271 images in the training set, featuring a diverse set of 29,672 facial images meticulously labeled by 40 annotators, encompassing both basic and composite expressions. The dataset’s richness is noteworthy, as it portrays significant diversities in terms of age, gender, ethnicity, head orientation, lighting conditions, and occlusions (such as glasses, facial hair, or self-occlusions) and post-processing alterations (including diverse filters and special effects). To further enhance the diversity of the dataset, ongoing experiments have incorporated additional data, broadening the model’s exposure to categories in which its training may be deficient.

The FERPlus dataset [22], short for Facial Expression Recognition Plus, stands out as a meticulously curated dataset tailored specifically for facial-expression recognition tasks. Distinguished by its emphasis on precision and granularity, FERPlus surpasses its predecessor, FER2013, through meticulous image relabeling efforts. It introduces eight primary expression categories: Anger, Disgust, Fear, Happy, Sad, Surprise, Neutral, and Contempt, offering nuanced insights into facial emotion depiction. Each image within the dataset is annotated with one or more expressions, providing a multifaceted view of emotional states. To enhance the diversity of the dataset and improve model performance, FERPlus incorporates sample augmentation techniques. By employing methods such as translation, rotation, and scaling, additional training samples are generated from the original images, enhancing the model’s resilience and adaptability. Such augmentation methodologies are prevalent in both research and practical evaluations within the realm of facial-expression recognition.

### 4.3. Performance Comparison

The performance of other state-of-the-art methods is compared with this paper’s method for RAFDB and FERPlus datasets with a uniform input image size of 224 × 224. Table 2 presents a comparison of the proposed method with other advanced methods in terms of accuracy, the number of parameters, and other factors.

After conducting a comparative analysis, the approach outlined in this paper demonstrates remarkable accuracy while utilizing a significantly lower number of parameters compared to current state-of-the-art methods, and the results on the RAFDB dataset will outperform those of recent lightweight methods. However, due to the conversion of the FERPlus dataset, which originally comprises 48 × 48 grayscale images, into 224 × 224 RGB images, the dataset lacks clarity and does not perform as well as the RAFDB dataset in terms of the recognition effect. Nonetheless, it still achieves satisfactory results.

### 4.4. Ablation Experiment

To assess the effectiveness of the ECA, GAM, ATS, and CB modules in the model, this paper conducts ablation experiments on the model based on the RAFDB and FERPlus facial expression datasets, and the outcomes of the ablation experiments are detailed in Table 3.

**Figure 8:**
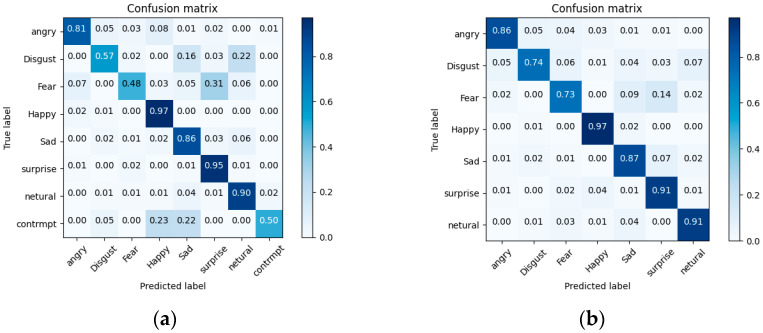
LiteFer confusion matrix. (**a**) LiteFer on FERPlus. (**b**) LiteFer on RAFDB.

As observed from the table, incorporating the CB module into the LiteViT component of the model presented in this paper significantly enhances the accuracy rate. Furthermore, the GAM attention mechanism proves to be more effective in improving accuracy compared to the ECA attention mechanism, although accompanied by a marginal increment in parameter count. Conversely, integrating the ATS module into the LiteViT component results in a slight decrease in accuracy. However, it notably reduces the model’s FLOPs and accelerates the training speed of the model. Figure 8 shows the confusion matrices of LiteFer on the FERPlus and RAFDB datasets.

**Figure 9:**
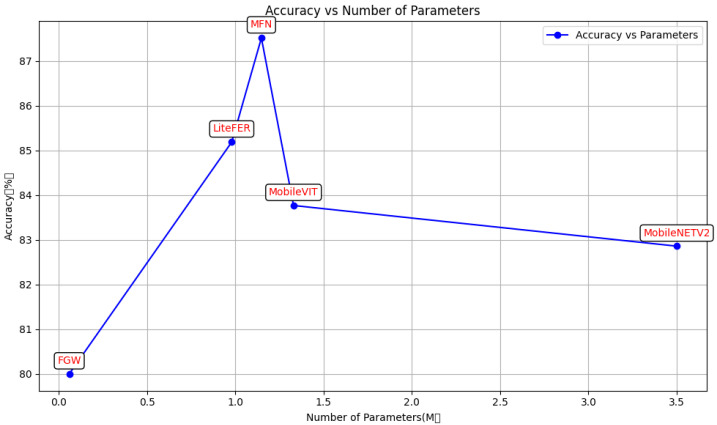
LiteFer average accuracy in RAFDB vs. other state-of-the-art methods.

**Figure 10:**
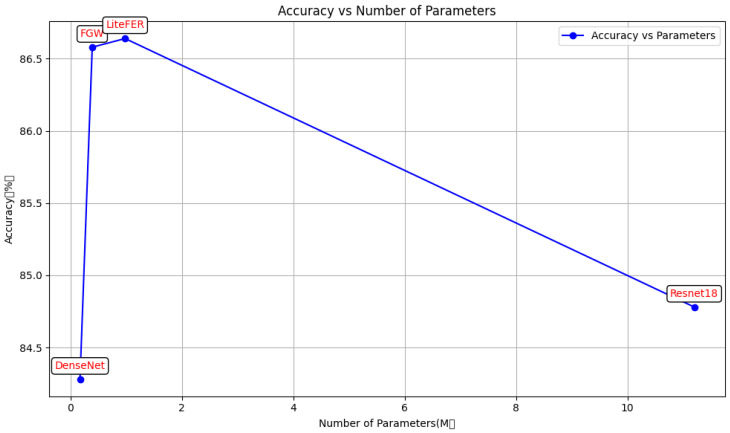
LiteFer average accuracy in FERPlus vs. other state-of-the-art methods.

Figure 9 presents a comparison between the proposed method and existing advanced methods on the RAFDB dataset. It can be observed that the proposed method achieves impressive results with a relatively lower number of parameters. Figure 10 shows a comparison of the proposed method with other advanced methods on the FERPlus dataset. Due to the input images used in our experiments being 224 × 224 pixels, which differs from the original size of the FERPlus dataset, the proposed method does not demonstrate a significant improvement over these advanced methods. However, it still achieves commendable results. Although the parameter count is significantly higher than that of FGW, the proposed method outperforms FGW in terms of accuracy and significantly surpasses MobileViT and MobileNetV2 in performance. The experimental findings underscore the efficacy of the GAM module attention mechanism and the fusion of convolution and vision transformer (ViT). A comparative analysis shows that the model proposed in this paper demonstrates commendable performance in facial-expression recognition. It is particularly well-suited for edge devices with limited computational resources and memory constraints due to its reduced model parameter count and computational footprint. The model maintains superior facial expression-recognition performance despite a decrease in parameters and computation. This is primarily attributed to the following reasons:

1. MobileNet Combined With the ViT Approach: This hybrid architecture enhances the model’s ability to learn local and global features simultaneously. By combining MobileNet’s capabilities for local learning with ViT’s expertise in global feature modeling, the model effectively focuses on a wider range of facial key regions while capturing subtle facial nuances.
2. GAM Attention Mechanism Module: The inclusion of the GAM module guarantees that the feature maps extracted by the MGAM module contain essential information without substantially increasing the parameter count. This mechanism allows the model to focus on crucial facial features, leading to improved recognition performance. Data augmentation techniques help address the problem of dataset imbalance, resulting in more robust training outcomes. Additionally, the integration of ATS adaptive sampling in ViT significantly reduces FLOPs, thereby accelerating the model’s training speed without compromising performance.
3. CB Module Integration: Adding the CB module at the end of the MLP in LiteViT enhances the performance of ViT without introducing additional parameters. This module helps in enhancing feature representation, leading to improved recognition accuracy.

## 5. Conclusions

In this paper, we introduced a method called LiteFer, an improved lightweight expression-recognition method based on MobileViT, and lightweight expression-recognition research, which provides new ideas and solutions for efficient emotion recognition in resource-constrained environments. Subsequent efforts should prioritize enhancing the model’s generalization capabilities, promoting practicality. The method presented in this paper achieves relatively advanced results among lightweight approaches; however, there is still a significant gap compared to the state-of-the-art results, such as Ada-DF [27] and DAN [28]. In cases where expressions are extremely ambiguous or difficult to distinguish, it can produce errors that are less likely to occur with larger models.

## Figures and Tables

**Table 2:** Comparison with state-of-the-art methods.

	Methods	Parameters	FLOPs	FERPlus	RAFDB
1	Resnet-18 [23]	11.18 M	1.82 G	84.78%	84.67%
2	MoblieNetV2	3.50 M	324.4 M	-	82.86%
3	MobileViT	1.33 M	261.6 M	-	83.77%
4	RAN [24]	11.2 M	14.5 G	89.16%	86.90%
5	MFN [25]	1.148 M	230.34 M	-	87.52%
6	DenseNet [26]	0.17 M	0.17 B	84.28%	-
7	TransFER [27]	65.2 M	-	90.83%	90.11%
8	LLTQ [28]	0.39 M	28 M	86.58%	
9	Ada-DF [8]	-	-	-	90.04%
10	DAN [29]	19.72 M	2.23 G		89.70%
11	FGW	0.06 M	-	79.36%	80.75%
12	LiteFer	0.98 M	218.3 M	86.64%	85.19%

**Table 3:** Ablation experiment.

	GAM	ECA	ATS	CB	Parameters	FLOPs	RAFDB	FERPlus
1		√			0.96 M	254.5 M	84.3%	84.9%
2	√				0.98 M	278.6 M	84.9%	86.4%
3	√			√	0.98 M	278.6 M	85.2%	86.8%
4	√		√	√	0.98 M	218.3 M	85.2%	86.6%

## Data Availability

The data that support the findings of this study are available from the first author, [X.Y.], upon reasonable request.
